# The pathogenicity and virulence of the opportunistic pathogen *Staphylococcus epidermidis*

**DOI:** 10.1080/21505594.2024.2359483

**Published:** 2024-06-13

**Authors:** Órla Burke, Merve S. Zeden, James P. O’Gara

**Affiliations:** Microbiology, School of Biological and Chemical Sciences, University of Galway, Galway, Ireland

**Keywords:** *Staphylococcus epidermidis*, infection, virulence, biofilm, host–pathogen interactions, therapeutic approaches

## Abstract

The pervasive presence of *Staphylococcus epidermidis* and other coagulase-negative staphylococci on the skin and mucous membranes has long underpinned a casual disregard for the infection risk that these organisms pose to vulnerable patients in healthcare settings. Prior to the recognition of biofilm as an important virulence determinant in *S. epidermidis*, isolation of this microorganism in diagnostic specimens was often overlooked as clinically insignificant with potential delays in diagnosis and onset of appropriate treatment, contributing to the establishment of chronic infection and increased morbidity or mortality. While impressive progress has been made in our understanding of biofilm mechanisms in this important opportunistic pathogen, research into other virulence determinants has lagged *S. aureus*. In this review, the broader virulence potential of *S. epidermidis* including biofilm, toxins, proteases, immune evasion strategies and antibiotic resistance mechanisms is surveyed, together with current and future approaches for improved therapeutic interventions.

## Introduction

Colonizing up to 80% of healthy humans,^1^ the frequent presence of *Staphylococcus epidermidis* and other coagulase-negative staphylococci (CoNS) on human skin demonstrates both its resilience to environmental extremes and capacity for host immune modulation,^2,3^ allowing it to evade detection by host defences. The skin can be a harsh environment for microorganisms, due to environmental factors such as variations in pH, temperature, and low moisture levels. Human epithelial surfaces also have multifaceted defence mechanisms mediated by the innate immune system, including production of a diverse array of antimicrobial peptides, sebum production, and the shedding of keratinocytes, all of which collectively inhibit colonization and infection by pathogenic organisms. Yet paradoxically, the skin still needs bacteria: formation of a healthy skin microflora can help prevent skin dysfunction, particularly well documented in the case of atopic dermatitis but also playing a significant role in stabilizing the microbial communities of healthy skin.^4,5^ Commensal inhabitants must balance strategies to survive desiccation, acid stress, and temperature fluctuations against the risks of activating the host immune system.

**Figure 1.**
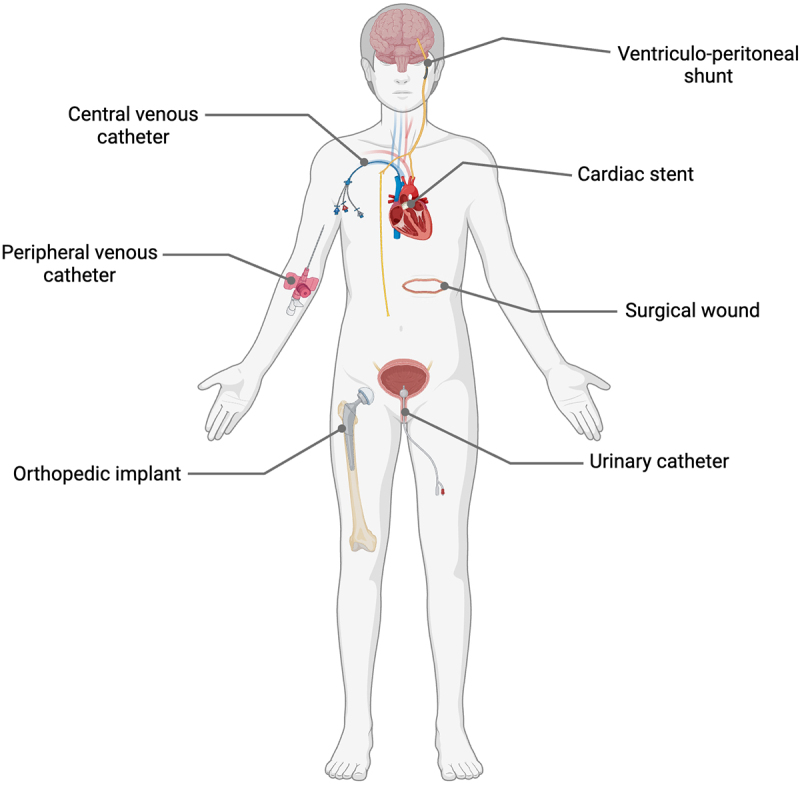
**Medical interventions including surgical wounds and various implanted medical devices associated with increased risk of** ***S.*** ***epidermidis*** **and other CoNS opportunistic infections.** Created with Biorender.com.

Due to its ubiquity, isolation of *S. epidermidis* in diagnostic samples taken during clinical infections was often dismissed as accidental contamination,^6^ before its emergence as an important pathogen in infections associated with implanted medical devices (Figure 1). Moreover, this pathogen is increasingly recognized as the sole causative factor in serious infections ranging from bacteraemia,^7^ sepsis,^8,9^ endocarditis, meningitis, and toxic shock syndrome,^10^ as well as superficial skin infections, ocular infections^11,12^ and the aforementioned infections associated with indwelling medical devices including shunts,^13^ catheters^14^ and prosthetic joints.^15^

The diverse array of infection vectors, including medical interventions and the patient’s own microflora, serve to emphasize the importance of *S. epidermidis*, through its ubiquity, as a challenging pathogen to eradicate.^16^ Moreover, to eliminate this commensal from epithelial surfaces may do more harm than good in the majority of cases, and this opportunistic pathogen continues to pose a significant health risk to vulnerable patients.^17^

Any discussion about *S. epidermidis* as a pathogen is invariably qualified by the comment that it lacks the diverse array of virulence factors found in *S. aureus*. Nevertheless, although this is true, we argue that it is important to consider what aspects of *S. epidermidis* pathogenicity are being overlooked when this microorganism is primarily categorized as a “common commensal” and not a ubiquitous opportunistic pathogen.

This review summarizes the pathogenicity of *S. epidermidis* infections including the molecular basis of biofilm-related and non-biofilm virulence determinants, as well as current treatment strategies and future directions for research on this remarkably persistent pathogen.

### Establishment of infection

**Figure 2.**
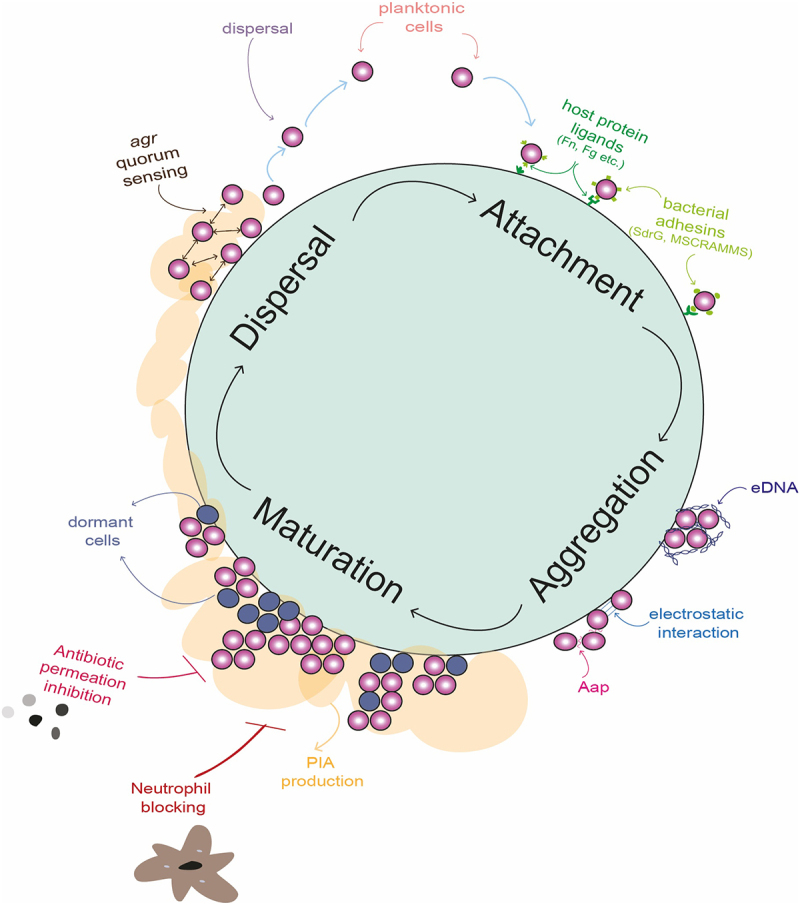
**Graphic illustration of the biofilm life cycle in *Staphylococcus epidermidis* including depictions of important mediators of attachment aggregation, maturation and dispersal stages of biofilm are depicted**. The attachment phase is characterised by the deposition of planktonic cells onto a biotic or abiotic surface. This phase is mediated by interactions between host ligands present on the surface, including fibrinogen (Fg) and fibronectin (Fn). Host proteins are then bound by bacterial surface adhesins, such as SdrG and microbial surface components recognizing adhesive matrix molecules (MSCRAMMs), which allow the bacterial cells to remain attached. The accumulation phase results in microcolonies of bacterial cells that self-associate using electrostatic forces and protein adhesins such as accumulation associated protein (Aap). Negatively charged extracellular DNA (eDNA) also surrounds the cells, attracted to the positively charged cell surface and resulting in a “sticky” mesh. A mature biofilm forms when polysaccharide intracellular adhesin (PIA) is excreted by *S. epidermidis*, forming profuse multi-layered structures. The biofilm matrix shields the bacteria from immune system cells, such as neutrophils, and provides protection from harsh environmental conditions such as desiccation, pH stress and the presence of antibiotics. Channels in the biofilm allow perfusion of nutrients. Within the mature biofilm, a significant proportion of cells remain metabolically dormant, rendering them tolerant to antibiotics that target metabolic processes. The dispersal of biofilm in *S. epidermidis* is co-ordinated by the action of the *agr* quorum sensing system, which secretes an autoinducing peptide when cell numbers increase to a critical level. In response to this, transcription of the *ica* operon, which governs the production of PIA, is reduced and individual cells are released from the biofilm structure. These planktonic cells are then free to disseminate in the host again.

As a frequent constituent of the human microbiome, *S. epidermidis* is generally considered to lead a commensal lifestyle on epithelial surfaces, such as the skin, nares, and the mucosa. However, *S. epidermidis* exploits breaches in the host’s cutaneous barriers, both through unintentional injury or surgical procedures in order to gain access to deeper tissues. Normally, the immune system is able to clear low-level infiltration by *S. epidermidis* cells, but immunocompromised individuals are susceptible to a spectrum of infections, from localized tissue inflammation to severe, systemic disease. Early establishment of infection involves bacterial adherence to surfaces, facilitated by microbial adhesins. This initial adhesion is a critical step, allowing the bacterium to evade clearance and establish a foothold within the host. Following successful adhesion, biofilm formation can begin (Figure 2).

### Common vectors of infection

Intravascular catheters, particularly central venous catheters and peripherally inserted central catheters, stand out as major sources of *S. epidermidis* infections. These devices provide an ideal substrate for bacterial adhesion and biofilm formation, leading to catheter-related bloodstream infections. More generally, CoNS are leading causative agents of bloodstream infections, representing up to 31% of reported cases.^18–21^ Prosthetic devices, including joint implants and cardiac devices, also represent significant sources of infection, causing chronic and difficult-to-treat biofilm-related infections. Periprosthetic joint infection (PJI) isolates are significantly more likely to be resistant to one or more antibiotics when compared to commensal isolates, and 44% of PJI isolates are from ST2, a sequence type associated with hospital-acquired highly resistant strains.^22^ Infection of cardiac devices such as pacemakers,^23^ as well as prosthetic heart valves^24, 25^ can necessitate removal of the device, or in refractive cases due to highly resistant strains, heart transplantation as salvage therapy.^26, 26^ In one study of bloodstream infections originating from cardiac assist devices, *S. epidermidis* was identified as the most common causative pathogen.^27^ In orthopedic *S. epidermidis* infections, which represent up to 43% of orthopedic device-related infections, aminoglycoside resistance, and strong biofilm-forming capabilities are associated with worsened patient outcomes.^28^

*S. epidermidis* is the causative agent in up to 60% of ventriculoperitoneal (VP) shunt infections,^29^ which are particularly challenging to treat due to poor antimicrobial drug penetration into cerebrospinal fluid (CSF). Post-operative infections of implanted VP shunts, which can affect up to 16% of patients, higher in neonates^29–32^ is accompanied by a mortality rate at 10%.^29^

Up to 31% of surgical site infections following spinal surgery are caused by *S. epidermidis*, and whilst *S. aureus* is a more frequent cause of these spinal surgery infections, methicillin resistant *S. epidermidis* (MRSE) was significantly more abundant than methicillin resistant *S. aureus* (MRSA) in spinal infections, which can limit treatment options.^33^ *S. epidermidis* was identified as the most common cause of latent surgical site infections after spinal fusion surgery, characterized by the slow development of clinical symptoms often mistaken for post-surgical back pain, which allowed the bacteria to become established at the surgical site and cause deeper infection, particularly inflammation of the vertebrae (spondylitis).^34^ Intracranial infection with MRSE may be associated with poor prognosis, even following vancomycin treatment.^35^ Isolates collected from infected sutures from a variety of surgical cases also implicated *S. epidermidis* as the most common pathogen.^36^ Surgical tools can also become contaminated with skin flora from either the patient or from surgical personnel during surgery, with *S. epidermidis* being the most commonly isolated species from laparotomy instruments,^37^ representing a vector of infection even when strict hygiene and aseptic technique are practiced.

Other vectors of implant-associated *S. epidermidis* infection include, but are not limited to, intrathecal pumps,^38^ breast prostheses and implants,^39–41^ intraocular lenses,^42, 43^ surgical mesh used for hernia repair,^44,45^ and bone-anchored hearing aids.^46^ Despite these risks, rigorous infection control measures, such as pre-operative decolonization, surgical site disinfection, and intra-operative infection control procedures can help prevent bacteria from breaching the skin during surgical procedures.^47–49^

### Chronic versus acute infection

*S. epidermidis* is typically responsible for chronic infections involving biofilm, rather than acute infections. However, examples of acute *S. epidermidis* infections include those associated with ocular implants^43, 50^ and knee arthroplasty.^51^ For the latter, poor response to treatment compared to non-staphylococcal infections can also lead to chronic infections.^52–55^ Prevention, or timely diagnosis and treatment of *S. epidermidis* infections is therefore of particular importance. The release of bacterial cells from biofilm into the bloodstream is noted as the primary mechanism of CoNS-related sepsis.^56^ Sepsis, a serious condition in which host immune response to bacteria in the bloodstream leads to systemic inflammation and potentially escalates to severe sepsis or septic shock, can in the case of *S. epidermidis* infection be viewed as an acute host response to a chronic bacterial infection.^57^

### Biofilm – the primary virulence factor

In a typical *S. epidermidis* infection scenario, biofilm formation begins with the transfer of bacteria from the skin onto a medical device during surgical implantation. The individual bacterial cells utilize their own surface protein adhesins to adhere to the implant, including interacting with host matrix proteins that coat the device. After adherent microcolonies are established, biofilm formation enters the accumulation phase, driven by the abundant production of polysaccharide intercellular adhesin (PIA)/poly-N-acetylglucosamine (PNAG), or protein adhesins that are covalently attached to the cell wall or associated with the cell surface. PIA forms a protective layer, enveloping the cells until mature biofilms begin to release planktonic cells, initiating a new cycle of dispersion. The role for different adhesins and biofilm mechanisms indicates that “biofilm” is not a uniform process, but a term encompassing various survival modes employed by *S. epidermidis* to adapt to dynamic and challenging host niches.

### Biofilm formation

Several reviews and articles have comprehensively covered *S. epidermidis* biofilm formation, structure, regulation, and disassembly in detail.^52,58–64^ Here, the role of the *ica* operon and the role of non-PIA biofilm factors such as extracellular DNA (eDNA) and key protein adhesins will be summarized to contextualize the interaction and importance of *S. epidermidis* virulence determinants with these biofilm constituents and regulators.

### Role of *ica* operon in PIA-dependent biofilm

Any review on *S. epidermidis* virulence cannot overlook the essential role of the *icaADBC* operon-encoded PIA in biofilm formation by many *S. epidermidis* isolates. The capacity of *S. epidermidis* to form biofilm strongly correlates with the establishment of chronic infections, and the accompanying morbidity and mortality to the host. Whilst *S. epidermidis* is also capable of *ica*-independent biofilm formation, PIA-mediated biofilm presents a uniquely profuse and resilient state of existence for staphylococci. Early research on “slime-producing” isolates of *S. epidermidis* identified the ability of what had previously been considered a harmless skin-dwelling commensal organism to persist within the host, turning simple implantation of medical devices into a procedure carrying significant risk for opportunistic infection by a ubiquitous skin commensal microorganism.^65–72^

**Figure 3.**
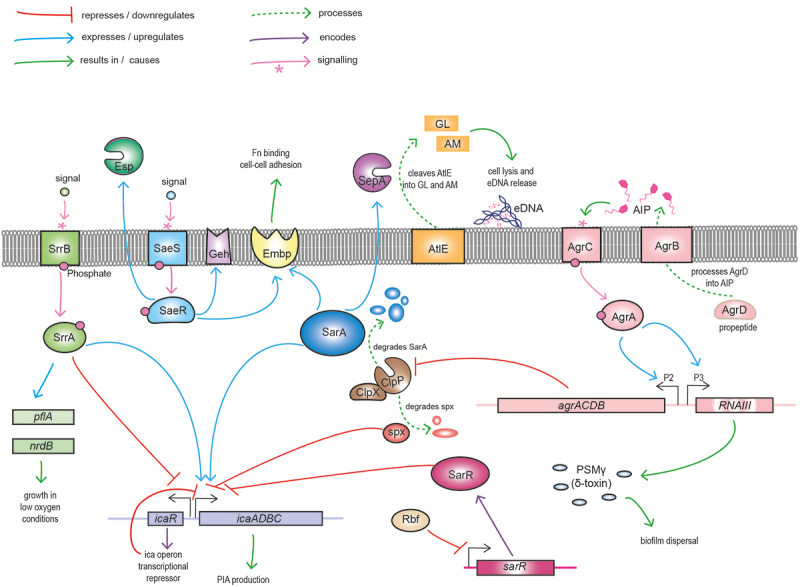
**Graphic illustration of major *S. epidermidis* regulators of growth, biofilm formation and virulence.** The *icaADBC* locus encodes the enzymes responsible for synthesis, export and deacetylation of polysaccharide intercellular adhesin (PIA), which serves as a major mediator of biofilm. The divergently expressed *icaR* gene encodes the major transcriptional repressor of the ica operon. In response to environmental cues activated SrrB phosphorylates its cognate SrrA effector promoting *pflA* and *ndrB* expression and growth under low oxygen conditions. SrrA binds to both the *icaA* and *icaR* promoters to differentially regulate biofilm under oxic and microaerobic conditions. Activation of the SaeS kinase leads to phosphorylation of the SaeR response regulator increasing fibronectin (Fn) binding and cell-cell adhesion through upregulation of GehD lipase and the extracellular matrix binding protein (Embp). SaeR also upregulates activity of the serine endopeptidase Esp, negatively impacting *S. aureus* colonisation and promoting *S. epidermidis* immune evasion through proteolysis of complement proteins. ClpPX is an ATP-dependent protease and chaperone system that degrades the global regulator SarA, resulting in repression of the repressor *icaR* and activation of *icaADBC* and biofilm. Rbf downregulates expression of *sarR*, which encodes a repressor of the *icaADBC* operon. The *agrD* gene from the *agrACDB* operon encodes a pro-peptide that is exported and processed by AgrB to release the autoinducing peptide (AIP) that constitutes that major quorum sensing system in staphylococci. In response to AIP, the AgrC sensor kinase phosphorylates the AgrA response regulator, thereby leading to activation of the P2 and P3 promoters and increasing expression of the *agrACDB* and RNAIII loci, respectively. RNAIII is the second effector molecule of the Agr system, functioning as an antisense RNA to control translation of target genes. The cytolysin phenol soluble modulin g (PSMg, δ-haemolysin), encoded by the *hld* gene located within RNAIII plays a role in biofilm dispersal. The Agr system also negatively regulates expression of the *clpP*-encoded protease, which degrades Spx, a negative regulator of *icaADBC*. SarA positively influences Embp expression facilitating cell-cell adhesion. Processing of the major autolysin AtlE by SepA to generate active amidase (AM) and glucosaminidase (GL) autolytic enzymes is important for eDNA release during the early stage of biofilm development.

The *ica* operon encodes a set of proteins responsible for PIA production, export, and function. The *icaA* and *icaD* genes encode transmembrane proteins that dimerize to form an N-acetylglucosaminyltransferase, responsible for PIA synthesis.^73^ The IcaB enzyme deacetylates PIA, introducing a positive charge necessary for its adherence to the cell surface, with *icaB* mutants markedly impaired in adhesion and colonization in the host, as well as being more vulnerable to phagocytosis.^74^ IcaC elongates the polysaccharide molecules, rendering them functional, whereas *icaC* mutants produce short, 20-residue oligomers in comparison to full-length PIA, which normally contains over 130 N-acetylglucosamine (GlcNAc) residues.^75^ The *icaR* gene located immediately adjacent to the ica operon encodes a transcriptional repressor centrally involved in the regulatory network that controls *icaADBC* transcription.^76–78^ Expression of *icaR* is in turn controlled by a complex network of proteins and transcription factors, including the alternative sigma factor σ^B^ and SarA, that combine to regulate the *ica* operon in response to metabolic, environmental, and host factors (Figure 3).^79^ Furthermore, transcription of the *icaADBC* operon does not precisely align with PIA production, revealing the importance of post-transcriptional regulation in the biofilm phenotype.

### PIA-independent biofilm

Approximately 30% of *S. epidermidis* isolates from non-superficial infections lack the *ica* operon.^80^ However, the absence of the *ica* operon does not preclude biofilm formation.^81^ PIA-independent or protein-mediated biofilm represents a second modality of biofilm formation. Protein-mediated biofilm relies on cell wall proteins that can bind to host ligands, as well as extracellular DNA (eDNA) released by autolysins that also enhances attachment and structure of biofilm. The spatial distribution of adhesins significantly influences biofilm architecture, for example, PIA encapsulates the cell, whereas protein adhesins localize to the membrane, resulting in different overall biofilm architecture which can give advantages in surviving in variant niches. PIA-producing isolates are more commonly isolated from sites exposed to shear force, e.g. the bloodstream and urinary tract, whereas PIA-independent biofilm in *S. epidermidis* represents the major mode of biofilm production in other prominent infection types, such as ocular infections.^80, 82^ PIA-dependent biofilm-forming strains are approximately twice as likely to cause prosthetic joint infections, compared strains capable of PIA-independent biofilm formation.^81^

### Aap

The cell wall-anchored (CWA) proteins like accumulation-associated protein (Aap) is the best characterized mediator of PIA-independent biofilm in *S. epidermidis*. Aap has several domains: a signal peptide, an N-terminal A domain, comprising A-repeats and L-type lectin domains, followed by a B-repeat region containing G5-E repeats. The C-terminal end contains an LPXTG motif that is covalently anchored to the cell wall by sortase.^83^ The A domain can be proteolytically cleaved by the metalloprotease SepA, exposing the B domain, which is then capable of self-association with other Aap B domains, to promote cell aggregation.^80^

The A-domain of Aap binds to host surfaces through the lectin domain, essential for adherence to host glycans. Heterophilic sugar binding by the lectin region of the A domain is also involved in initial staphylococcal cell–cell contact early in biofilm formation, before homophilic B-repeat bonds allow for tighter binding. Homophilic interactions between adjacent cells involve the G5-E domains binding to one another. Notably, the B-repeat domains exhibit amyloid fibril formation, forming “rope-like” structures within the biofilm matrix, a process dependent on zinc equilibrium.^80, 84^ Single-molecule experiments have revealed the extraordinary mechanical strength of Aap homophilic bonds, with forces reaching up to 1,000 pN – close to the bond strength of covalent chemical bonds^85^ reinforcing their role in forming highly adhesive and cohesive biofilms (Figure 2). Intriguingly, the cleavage patterns of Aap vary between strains, with a 2022 study by Wang et al. finding that strain CSF41498 has lower rates of Aap processing resulting in more intact Aap when compared to strain 1457, which has increased cleavage of Aap.^86^

### Extracellular matrix binding protein (EmbP)

Embp is a large, membrane bound protein involved in adhesion. Whilst EmbP plays a moderate role in adhesion to fibronectin (Fn) under low flow conditions, the “Velcro-like” binding that occurs between multiple repeats within EmbP and the repeats exposed in the fibrillated, deposited form of Fn was shown to be crucial to *S. epidermidis* binding to surfaces under high shear conditions, for example on an implanted intravenous catheter.^87^ Embp expression is highly induced when adherent cells, such as those in a biofilm, are subjected to osmotic stress indicating that Empb may be more important for survival of *S. epidermidis* by promoting cell–cell adhesion under the high-salinity conditions found on the skin, rather than as a factor in invasion and adhesion during infection. However, given the role of Embp in binding to Fn, osmotic stress induced Embp expression should enhance adherence to the host extracellular matrix proteins and enhanced biofilm formation in the infection niche.^88^

### Biofilm associated protein (Bap)/bap homolog protein (Bhp)

The first report of a PIA-independent mechanism of biofilm formation in staphylococci identified Bap.^89^ This 239-kDa sortase-anchored surface protein contributes to the initial attachment and accumulation phases of biofilm.^90^ Unlike Bap in *S. aureus*, which is associated primarily with bovine mastitis isolates, Bap in *S. epidermidis* is commonly found in mastitis isolates from several veterinary species.^90^ Bap consists of several repeated domains, referred to as Bap repeats, which are responsible for its adhesive properties and the formation of cell-to-cell interactions within the biofilm. Bhp is a protein with significant homology and similarity to Bap,^90^ which is also found in non-animal derived isolates, including in MRSE isolates from clinical infections.^90^ Bhp has an N-terminal export signal, followed by an A-domain and a region of tandem repeats that shares 45–57% identity with *S. aureus* Bap tandem repeats.^91^ Bhp expression is highest during early exponential phase and decreases in stationary phase.^91^ Serum reactivity to Bhp was not commonly detected in patients with *S. epidermidis* infections, suggesting that Bhp is only weakly immunoreactive or not highly expressed during infection, although other cell-wall proteins like SdrF exhibited a similar response.^91^ Found in both commensal and invasive strains,^92^ and in up to 15% of blood culture isolates,^93^ the role of Bhp in *S. epidermidis* biofilm formation remains unclear, and whilst it shares considerable similarity with Bap, it is important not to over-extrapolate from the small amount of available literature on Bhp.

### eDNA and cell lysis

Extracellular DNA (eDNA) is released from lysed bacterial cells, either through breakdown of dead cells or by the activity of autolysins.^94^ eDNA has multiple properties that make it a valuable biofilm constituent. eDNA is associated with the initial attachment phase to abiotic surfaces.^95–97^ Strands of eDNA create a scaffold that helps maintain the overall architecture and resilience of the biofilm (Figure 2).^98^ eDNA also acts as a barrier against host immune defences by binding and sequestering antimicrobial peptides.^99, 100^

*S. epidermidis* possesses two autolysins, the major autolysin AtlE and a secondary autolysin Aae, both having dual roles as both autolysins and adhesins. AtlE consists of a signal peptide and a propeptide, followed by amidase (AmiE) domain linked by three direct repeats (R1, R2, R3) to a glucosaminidase domain at the C-terminus. Proteolytic processing occurs by an unidentified protease, resulting in removal of the N-terminal signal peptide and the propeptide after secretion.^101,102^ After further processing by the metalloprotease SepA,^101^ the amidase domain retains two of these repeats, and the glucosaminidase domain retains the third. Each repeat contains a glycine-tryptophan (GW) module. The amidase domain is anchored to the cell wall by the R1 and R2 repeats and is required to cleave peptidoglycan during cell division.^103,104^ Evidence from the functionally interchangeable Atl protein in *S. aureus* indicates that presence of WTA on the mature cell wall but not the newly created septum discourages AtlE binding during division, encouraging enzymatic separation of daughter cells.^104,105^

Binding and internalisation by host cells are mediated by the AmiE domain, which interacts with the host heat shock cognate protein Hsc70 directly and with α5β1 integrin via fibronectin bridging.^106, 107^ Vitronectin binding activity is seen with the full-length protein, and to a lesser extent with each of the catalytic domains.^102^ Deletion mutants of *atl* are defective in biofilm formation^94^ and have decreased virulence in a rat catheter model.^108^ eDNA is found in microcolonies during the initial adherence phase of biofilm accumulation. DNase I is capable of dispersing early-stage biofilms, but ineffective at removing mature biofilm.^94, 109^

Aae is a more recently identified autolysin.^110^ The N-terminal domain of Aae consists of an LysM domain, implicated in peptidoglycan recognition and binding, made up of three direct repeats. Unlike other bacterial species where it is located at the C-terminal end, the N-terminal position of the LysM domain in staphylococci may allow Aae to associate with the cell wall in the absence of a specific cell wall anchor. Like AtlE, Aae also has adhesive properties and can bind fibrinogen, fibronectin and vitronectin in a dose-dependent manner.^110^

The link between PIA production and eDNA in biofilm matrix is complex, though the overall trend is positive, with strong PIA production being associated with increased levels of eDNA. PIA may play a role in retaining eDNA within the biofilm structure.^98^ Thus, eDNA appears to play a role in both PIA-dependent and PIA-independent biofilm development.

Interestingly, several eDNA-binding proteins have been described in the matrix of *S. aureus* biofilms, many of which were previously not known to bind DNA,^96^ forming positively charged surface anchors that stabilize the electrostatic net of eDNA in the matrix.^96^ Several of these proteins have homologues in *S. epidermidis*, including CopL, which is associated with the COMER-like pathogenicity island found in the hospital-associated MRSE lineage ST2.^111^ Furthermore, the glucosaminidase region of *S. aureus* Atl is also known to bind DNA.^112^ This raises the intriguing possibility that there remain uncharacterized eDNA and protein interactions in *S. epidermidis* biofilms. Regulation of eDNA release is mediated through SarA, which indirectly negatively regulates AtlE by downregulating SepA, responsible for the proteolytic cleavage required for the bacteriolytic function of AtlE.^101^

A small regulatory RNA RsaE was recently identified that positively regulates both *icaADBC* expression and eDNA release. RsaE is expressed differentially throughout *S. epidermidis* biofilm, and interestingly is not present in all *icaADBC*-positive strains. Regulation of PIA production is mediated through RsaE interaction *icaR* mRNA, whilst the effect on autolysis occurs due to negative regulation the *lrgA*-encoded antiholin protein that prevents cell lysis by the holin CidA.^113^ In *S. aureus*, eDNA release by CidA contributes to the function of biofilm,^114^ suggesting that it may play a similar role in *S. epidermidis.*^113^ In *S. epidermidis* small colony variants (SCVs), imbalance between CidA and LrgA expression results in increased eDNA abundance in biofilm.^115^

## Non-biofilm virulence determinants

Despite being commensals, *S. epidermidis* strains are capable of expressing several enzymes and toxins, which collectively appear to play a protective role underpinning the characteristic resilience of this organism.

### Lipases

Lipase-mediated hydrolysis of lipids can serve as a source of nutrients for *S. epidermidis*. Interestingly, the glycerol ester hydrolase (*geh*) lipases of staphylococci have a secondary role as surface-anchored adhesins. *S. epidermidis* has two known *geh* lipases, GehD and GehC, under the control of the *agr* quorum sensing system.^116^ GehD is a collagen-binding MSCRAMM, which despite lacking an LPXTG motif is localized to the cell wall^117^ (Figure 3).

### Superantigens and enterotoxins

Staphylococci produce multiple superantigens implicated in toxic shock syndrome (TSS) including TSS toxin 1 (TSST-1), and staphylococcal enterotoxins B and C.^118, 119^ Superantigens bind to Major Histocompatibility Complex class II (MHC II) receptors, precipitating interactions with T-cells and inciting a cascade of events leading to heightened cytokine production, marked inflammation, and the onset of TSS symptoms such as fever, hypotension, vomiting, and confusion.^120^ Menstrual TSS is caused almost exclusively by TSST-1, whereas staphylococcal enterotoxin B and C as well as superantigens from group B, C, and G streptococci are also known to be involved in non-menstrual TSS.^121^ CoNS are generally considered to lack superantigens as they lack TSST-1, however, they may possess staphylococcal enterotoxins.^10^ Up to 95% of *S. epidermidis* isolates from blood cultures possess at least one staphylococcal enterotoxin.^122^

Staphylococcal enterotoxins (SEs) exhibit a remarkable tolerance to both heat and pH stress, as well as protease degradation, maintaining their integrity in the harsh environment of the gastrointestinal (GI) tract^123^. Staphylococcal food poisoning (SFP) from SEs is accompanied by a rapid onset of symptoms, including nausea, emesis, abdominal pain, diarrhea and GI tract inflammation and damage.^123^ Whilst reports of disease caused by superantigen- and enterotoxin-producing *S. epidermidis* isolates are limited to sporadic cases in the literature, a small minority of strains do possess and express these virulence factors, which can be clinically important. An early case of SEA-producing *S. epidermidis* was implicated in a major outbreak of SFP in the 1960s.^124^ A novel C-type enterotoxin (SEC) from *S. epidermidis* was shown to induce high levels of cytokine secretion, including IL-2, −4, −6, −8, −10, IFN-γ, TNF-α and GM-CSF.^125^ This massive release of multiple cytokines in response to MHC II binding is characteristic of superantigenic activity.^120^ Production of IL-6 by lymphocytes in response to SEC is notable as IL-6 is a “pro-sepsis” cytokine that contributes to the systemic inflammation seen in septic shock.^125^ Interestingly, the *S. epidermidis* homologue of C-type enterotoxin induced higher secretion of IL-6 when compared to its *S. aureus* homologue.^125^ SEC and another enterotoxin, SEL are often carried on the same *S. epidermidis* pathogenicity island^126^ and oral administration of SEL and SEC in a mouse model was shown to cause oedema and cytotoxicity in the GI tract.^127^

A recent case of *S. epidermidis* toxic shock syndrome was reported in which novel PCR assay was used to detect multiple staphylococcal enterotoxins including TSST-1 in blood plasma.^10^ Interestingly in this patient, *S. epidermidis* was only isolated from a urinary culture and not blood cultures.^10^ TSST-1 is also found infrequently in veterinary isolates of *S. epidermidis*, and its horizontal gene transfer (HGT) from CoNS to *S. aureus* has been widely studied.^128^ Notably one paper described likely HGT of a pathogenicity island in the opposite direction: from *S. aureus* to two virulent strains of *S. epidermidis*, allowing for expression of an enterotoxin. This was previously considered to be controversial due to the presence of strong restriction barriers in *S. epidermidis.*^128^ The presence of superantigens in *S. epidermidis* is uncommon, and these authors concluded that enterotoxin produced by these strains may have contributed to septic shock in the patients from which they were isolated.^128^ Overall, while reports of *S. epidermidis* toxic shock and enterotoxigenicity remain rare in the literature, it nevertheless appears that individual *S. epidermidis* strains may have the capacity to produce both superantigens and enterotoxins.

## Proteases

### Serine protease (Esp)/glutamyl endopeptidase (GluSE)/SspA

The serine endopeptidase Esp is a secreted protease that belongs to the glutamyl endopeptidase I family, sharing about 50% identity with the better-characterized *S. aureus* V8 protease.^129^ Esp is ubiquitously found in *S. epidermidis* strains, in which it is also known as SspA and GluSE.^17, 130^ Production of Esp is associated with adherent, biofilm-like growth rather than planktonic growth.^130^ Esp contributes to the ability of *S. epidermidis* to compete with *S. aureus*^131^ by cleaving the B-domain of the *S. aureus* Aap protein required for the attachment phase of *S. aureus* biofilm formation, as well as degrading fibrinogen, fibrin.^80, 132, 133^ Elastin, fibronectin, and type I collagen,^134^ which all play a role in *S. aureus* interaction with host cells.^80, 132–134^ Esp is also involved in evading host immune defences by degrading complement protein C5.^132, 134^ Finally, Esp is a significant virulence factor in ocular infections,^135^ which is important given that *S. epidermidis* is a leading cause of keratitis, blepharitis, conjunctivitis, and endophthalmitis.^135^

### Metalloprotease (SepA)/aureolysin

As noted above SepA processes Aap, cleaving off the A-domain, and sometimes the lectin domain to leave the B-domain.^136^ The regulator SarA controls expression of *sepA*, which is not observed for the other proteases (Figure 3).^80^ A notable role for SepA is in promoting bacterial survival following neutrophil phagocytosis, contributing to immune evasion and maintenance of infection.^137^ SepA has also been reported to degrade AMPs and process AtlE^132^ (Figure 3). SepA-mediated degradation of the anionic human AMP dermcidin is important for survival on the skin and also notable given that the standard staphylococcal defence mechanisms against CAMPs, such as altering the bacterial cell wall charge via D-alanylation of wall teichoic acids, are ineffective against anionic dermicidin.^138^

### Extracellular cysteine protease (EcpA)

EcpA is a secreted cysteine protease with gelatinase and collagenase activity.^35^ EcpA is negatively regulated by the protease inhibitor EcpB, also known as staphostatin A, and positively regulated by *agr* system.^132, 139^ Secretion of EcpA damages the epithelial barrier by breaking down host barrier proteins, primarily desmoglein-1.^35^ This breakdown in dermal integrity increases disease severity in atopic dermatitis.^35^ EcpA is homologous to SspB in *S. aureus* which is known to degrade the LL-37 epithelial antimicrobial peptide.^132^ Cau et al. recently showed that *S. epidermidis* EcpA also degrades LL-37, and increased expression of IL-6, IL-8, TLSP, IL1-α and IL-1β in host keratinocytes, promoting inflammation.^140^ Autoinducing peptides (AIPs) from other CoNS species, such as *S. hominis*, can decrease expression of EcpA and the presence of a healthy skin flora may therefore inhibit *S. epidermidis*-mediated skin damage in patients with AD.^140^

### Epidermidin leader peptide processing serine protease

The extracellular epidermin leader peptide processing serine protease (EpiP) cleaves the propeptide EpiA, generating the functional lantibiotic form of epidermin.^141^ Lantibiotics such as epidermin have broad antibacterial activity microorganisms, and consequently self-immunity proteins are required to prevent ill effects from the production of these molecules.^142^ Whilst the primary function of epidermin is in defence, EpiP is also involved in cleavage of host proteins such as collagen and casein,^132, 143^ resulting in breakdown of connective tissue. Furthermore, the *Streptococcus pyogenes* homolog of EpiP impairs neutrophil recruitment, raising the possibility that *S. epidermidis* EpiP has a similar function.^143^

### Cytolysins

The phenol soluble modulins (PSMs) are the major cytolysins in *S. epidermidis*^144, 145^ and comprise a family of small amphipathic, α-helical peptides that also serve as effectors of staphylococcal biofilm structuring and dispersal both *in vitro* and *in vivo.*^144, 146, 147^ Clinical PJI-associated biofilm forming strains are less likely to produce PSMs, potentially due to the dispersal effect PSMs have on established biofilm.^148^

*S. epidermidis* produces 6 types of PSMs: PSMα, PSMβ1, PSMβ2, PSMγ (also known as δ-toxin), PSMδ, and PSMε, and may additionally possess PSM-*mec*.^149, 150^ PSMγ also known as δ-haemolysin is encoded by the *hld* gene located in the RNAIII locus of the *agr* operon.^122, 148^ α-PSMs are small, approximately 20–25 amino acids in length and are associated with cytolysis of immune cells and induction of neutrophil chemotaxis.^151^ β-PSMs, characterised by their larger (45 amino acid) size, disrupt the biofilm matrix by interfering with the non-covalent forces involved in cell-cell interactions.^152^ At lower concentrations of β-PSMs, this allows for the formation of channels, a process essential for the delivery of nutrients to deeper biofilm layers, whereas higher levels of the PSM result in dispersal of biofilm, allowing planktonic cells to be released.^152, 153^ The chromosomal locations for the genes for α and β PSMs are found separate from PSMγ/*agr.*^148^ β-PSMs genes occur in tandem on the chromosome, with two identical genes next to each other.^148^ The *psm-mec* gene is located in the SCC*mec* mobile genetic element.^154^ Independent of the PSM-mec peptide, the *psm-mec* RNA acts as an independent regulator of RNAIII.^155^ PSM-*mec* influences the virulence of *S. epidermidis* in sepsis and is highly inflammatory and induces neutrophil chemotaxis.^154^

Beyond the PSMs, β-toxin, encoded by the *hlb* gene, is a sphingomyelinase, which in *S. aureus* can lyse erythrocytes^156^ and kill proliferating human T-lymphocytes,^157^ but in *S. epidermidis* has a protective role in promoting production of ceramides important for skin integrity.^158^

## Regulation of virulence

### Quorum sensing

Staphylococcal quorum sensing is primarily mediated by autoinducing peptides (AIPs) that are secreted from the cell and recognised by their cognate receptors.^159^ Quorum sensing regulates *S. epidermidis* virulence in a cell-density dependent manner, triggering changes in exotoxin secretion and adherence factors as well as regulating formation and dispersal of biofilm.^160–163^

### Accessory gene regulator (Agr) system

The Agr system, encoded by the *agrABDC*-RNAIII locus, is a quorum sensing system that plays a key role in regulating virulence in *S. epidermidis* and is regulated in a density-dependent manner^164^ The *agr* system produces two effector molecules. The first, AgrA, is a cytoplasmic response regulator that is phosphorylated by AgrC, a membrane-bound histidine kinase, in response to the *agrD*-encoded autoinducing peptide (AIP) after a threshold concentration is reached.^165, 166^ The AgrD pro-peptide is processed and exported by AgrB, in tandem with the signal peptidase SpsB.^116, 167^ AgrA also controls expression of a secondary effector molecule, RNAIII.^163^ RNAIII is a 510 nucleotide non-coding RNA molecule that controls protein production by acting as an antisense RNA, with the long 5’ untranslated region (UTR) of RNAIII when binding to the mRNA product of target genes, impairing translation.^168–171^

In *S. epidermidis*, three different *agr* groups have been described, (I, II, III) based on polymorphisms identified within the *agrC*, *agrB* and *agrD* genes that result in different types of AIP production.^116, 172, 173^

AgrA autoregulates its own expression, and also regulates RNAIII transcription^166^ (Figure 3). Agr controls expression of many *S. epidermidis* virulence genes, including *geh* lipase and *ecp* cysteine protease.^160^ An active *agr* system is strictly required for the expression of PSMs.^144, 148, 174^ PSM production regulated by the AgrA/AIP circuit occurs primarily in the late-log phase of growth, which coincides with maximal *agr* activity.^160,166^

As noted above, the *hld* gene encoding for production of PSMγ (δ-haemolysin) is encoded within the transcript for RNAIII.^170^

The cysteine protease EcpA, which triggers production of pro-inflammatory cytokines and disrupts the epithelial membrane is positively regulated by the *agr* system.^140^ In the absence of a functional *agr* system, no activation of host TNF-α and HIV1-LTR is observed, and neutrophil chemotaxis was reduced.^175^ The presence of the *agr* system is also associated with resistance to cathelicidin and β-defensin AMPs.^176^

The *agr* system plays a central role in controlling the virulence of *S. epidermidis* under both biofilm and planktonic conditions. A biofilm contains a variety of niches with cells in different metabolic and physiological states, and so the ability of *S. epideridis* to tightly control the regulation of biofilm dispersal as well as the induction of inflammation in a host is critical for establishment and maintenance of disease (Figure 3).

### LuxS/Autoinducer-2 quorum sensing

Autoinducer-2 (AI-2) is the signal sensed by a proposed cross-species quorum sensing system present in both Gram-positive and Gram-negative bacteria, originally identified in *Vibrio harveyii.*^177^ AI-2 is derived from a precursor molecule produced by LuxS. The characterization of AI-2 signalling in staphylococci remains somewhat unresolved, with separate papers describing a role in both positive^178^ and negative regulation^179^ of biofilm. Furthermore, the positive regulation of biofilm was strain dependent with RP62A showing decreased biofilm in a *luxS* deletion mutant, whereas similar experiments with CSF41498 and clinical isolates revealed no impact on biofilm^178^ Addition of exogenous AI-2 was shown to differentially regulate genes involved in glycometabolism and amino acid, nitrogen, and nucleotide metabolism.^178^ PSM production was also increased in the presence of AI-2, independent of *agr* regulation.^178^ Whilst its role remains to be fully elucidated, the available evidence suggests that when AI-2 quorum sensing is active in *S. epidermidis*, it plays a strain-dependent role in modulation of biofilm formation and metabolic regulation.

## Global regulators

### Alternative sigma factor σ^B^

σ^B^ is centrally involved in the environmental regulation of *icaADBC* expression and biofilm production^180^ and controls the transcription of >200 staphylococcal genes.^181^ Under normal growth conditions σ^B^, which is encoded by the *sigB* gene in the *rsbUVWsigB* operon, is bound to the anti-σ factor, RsbW and consequently inactive.^79, 182^ The RsbU phosphatase is activated by environmental stress leading to dephosphorylation of RsbV, which binds to RsbW, thereby releasing σ^B^ to engage with the core RNA polymerase and up-regulate transcription of genes with σ^B^-dependent promoters.^79, 183^ However, neither the *icaR* nor *icaADBC* promoters are σ^B^-dependent.^184^ Furthermore, σ^B^ controls *ica* operon expression by indirectly regulating expression of *icaR.*^79, 180, 183^ Thus stress-induced activation of σ^B^ leads to repression of *icaR* transcription, via an unidentified intermediary, and the consequent activation of the *ica* operon. σ^B^ also plays a role in the stability of mature *S. epidermidis* biofilm, contributing to persistence in a rat catheter infection model.^185, 186^

### CodY

The CodY global regulator in *S. epidermidis* acts as a sensor of nutrient availability to coordinate gene expression in response to nutrient fluctuations. The CodY regulon consists of hundreds of negatively regulated genes that contain CodY boxes, typically located within their promoter regions.^187^ CodY binding interferes with RNA polymerase interaction with the promoter, downregulating expression. When nutrient levels are high, CodY is activated by its ligands, branched-chain amino acids (BCAAs) or GTP, and binds to the CodY box, resulting in transcriptional repression of target genes^160, 188^ In contrast, under conditions of nutrient limitation, dissociation of CodY from the CodY box leads to de-repression of target gene expression.

In staphylococci, CodY is particularly associated with negative regulation of virulence.^187^ In *S. aureus*, CodY negatively regulates the *ica* operon, leading to reduced production of PIA and impaired biofilm formation.^187, 189^ The role of CodY in *S. epidermidis* biofilm formation has yet to be clearly elucidated, though a possible role in induction of cell dormancy during stationary phase and in biofilms has been shown, and a role in regulation of *agr* has been clearly demonstrated.^190–192^

Other staphylococcal virulence factors regulated by CodY include secreted extracellular proteases like SspA and AtlE, as well as the *geh* lipase, the *hla* α-haemolysin and the surface protein SasG.^193^ CodY also indirectly regulates the global regulator SarA by repressing the expression of *rot*, which encodes a repressor of *sarA*. Reduced levels of Rot relieve SarA repression, leading to increased expression of SarA-regulated virulence genes^194^ (discussed in more detail below). In staphylococci, expression of CodY itself is negatively regulated by the *agr* system^160^ and in turn, strongly represses expression of *agr* through an unknown intermediate, revealing the complexity of this network of interconnected virulence regulators. Whilst these targets have been primarily identified in *S. aureus*, *S. epidermidis* homologues (*ica,*^195^ *sspA,*^132^ *atlE,*^196^ *geh,*^196^ *sasG*/*aap,*^197^ *sarA,*^198^ *agr*^165^) may also be regulated by CodY.

### Sar family regulators and Rbf

Sar family regulators are characterized as winged-helix family of transcriptional regulators with conserved DNA binding domains that typically bind to the promoter regions of target genes, up- or down-regulating their expression.^199^

SarA is particularly associated with the control of biofilm and virulence gene expression including a central role in the positive regulation of PIA-mediated biofilm formation (Figure 3). IS*256* insertional inactivation of *sarA* impaired biofilm formation in the *ica*ADBC-positive strain RP62A, which was reversed by clean excision of the insertion sequence.^180^ SarA can bind to the *icaA* promoter, activating transcription of *ica* and production of PIA,^198^ which implied that SarA controls biofilm in an *agr-i*ndependent manner.^198^ The *S. epidermidis sarA* mutant also showed decreased extracellular protease activity.^198^ SarA may also play a role in switching between polysaccharide- and protein adhesin-mediated biofilm formation.^80^ A transposon inactivation of *sarA* increased biofilm production in *ica*-negative *S. epidermidis* 1585 due in part to upregulation of the *sarA*-repressed Embp biofilm protein and SepA metalloprotease (which processes AtlE and Aap)^80, 101^ (Figure 3). Regulation of *sarA* itself remains incompletely understood and is driven by three promoters designated SarP1, SarP2, and SarP3, of which SarP1 is σB-dependent. σB regulates *ica* operon transcription in *S. epidermidis* via indirect regulation of the *icaR* repressor.^184^ SarA positively regulates the *ica* operon by directly binding to the *ica* promoter, independently of σB^184^ (Figure 3).

SarZ is involved in both the initial attachment and accumulation phases of biofilm formation, as well as regulating the expression of serine protease and *geh* lipase genes.^77^ Interestingly, deletion of the *sarZ* gene resulted in β-haemolysis, a phenotype not typically observed in *S. epidermidis.*^200^

SarX also positively regulates the expression of the *ica* operon though direct interaction with the *ica* promoter, resulting in increased production of PIA.^201, 202^ SarX also regulates the *agr* locus, but the effect on biofilm production is predominantly due to *ica* expression.^201^

### Rbf

The AraC/XylS-family transcriptional regulator *rbf* (Regulator of biofilm formation) gene is located immediately upstream of *sarX* and positively influences biofilm in both *S. aureus* and *S. epidermidis.*^203, 204^ Rbf does not interact with the *ica* operon promoter region of *S. epidermidis* demonstrating that it controls PIA-mediated biofilm indirectly.^205^ Electrophoretic mobility shift assays also showed that Rbf bound specifically to the *sarR* promoter but not to the promoters of the *sarX*, *sarA*, *sarZ*, *spx*, and *srrA* genes.^205^ SarR was subsequently shown to be a repressor of *ica* operon transcription revealing that Rbf controls biofilm by downregulating expression of the *sarR* repressor.^202, 205^ These findings further reveal the complexity of *S. epidermidis* PIA regulation as well as the importance and sophisticated fine tuning of this phenotype.

## Two-component signal transduction systems

### *S.* *aureus* exoprotein expression regulator (SaeRS)

SaeRS has been extensively studied in *S. aureus,*^206^ but the cognate *S. epidermidis* system has notable differences, including the absence of the SaeP and SaeQ accessory proteins involved in *S. aureus* response to neutrophils.^207, 208^ Virulence genes under the control of SaeSR include the *geh* lipase gene and the *esp* serine protease gene (Figure 3).^207^ In *S. aureus*, SaeS senses human neutrophil peptide 1 (HNP1), but not in *S. epidermidis.*^206^ SaeRS also contributes to inflammation in a mouse catheter infection model, with increased proliferation of polymorphonuclear neutrophils, which occurs independently of PSM production.^207^ Deletion of SaeR results in impaired nitrate utilization and decreased expression of genes involved in anaerobic growth, implicating the SaeRS system in mediating the switch between aerobic and anaerobic growth^207^. However, *saeR* deletion does not impact *ica* transcription levels, and it is suggested that the differential regulation of virulence genes in a *saeR* mutant may be indirect, due to the effects on metabolism^209^. Activation of SaeRS decreases autolysis by downregulating transcription of *aae* and *atlE* (Figure 3), which impacts cell survival and viability during biofilm and planktonic growth.^209^

#### Staphylococcal respiratory response protein (*srrAB*)

Another two-component system that regulates both survival and virulence of *S. epidermidis* is SrrAB (Figure 3). Unlike its cognate system in *S. aureus*, which is only expressed only under microaerobic conditions,^210^ SrrAB in *S. epidermidis* is active under aerobic conditions and upregulated under low-oxygen conditions.^211^ SrrAB regulates both initial adherence and subsequent biofilm matrix production, as well as overall growth and fitness under both aerobic and microaerobic conditions.^211^ Specifically, under aerobic conditions, the phosphorylated response regulator SrrA, which binds to both the *icaA* and *icaR* promoters, positively regulates *icaADBC* expression while simultaneously downregulating *icaR* expression, resulting in increased PIA production and biofilm formation.^211^ Under microaerobic conditions, deletion of *srrA* results in a significant growth defect, and transcription of both *icaR* and *icaADBC* is downregulated.^211^ SrrA also positively regulates the *qoxBACD*-encoded respiratory chain terminal oxidase under oxygen-replete conditions. SrrA controls expression of *pflBA* and *nrdD* during microaerobic growth, also through direct interaction with the promoter regions of these genes.^211^ The ability of SrrAB to modulate growth and biofilm production in oxygen-replete and oxygen-limited environments highlights the capacity of *S. epidermidis* to adapt to a wide range of environmental conditions and is likely to contribute to its pathogenicity.

#### GraXSR/VraFG

The GraXSR (also referred to as ApsRSX) two-component system and neighboring VraFG efflux system are involved in resistance to cationic AMPs (CAMPs).^212^ GraS is a membrane-associated histidine kinase which works together with GraX and the response regulator GraR.^213^ GraXSR is known to modulate the activity of VraFG, a permease and ATPase respectively, and all five proteins function together as a signal transduction complex.^213^ The function of GraX remains unclear, but it may act to potentiate the signal transduction of the GraXRS/VraFG complex.^213^ A nine amino acid extracellular loop of the GraS protein senses CAMPs.^213^ One model suggests that in *S. epidermidis*, CAMP interaction with the extracellular sensor of GraS dislodges the guard loop of VraG from the membrane, leading to a change in VraG conformation that could positively modulate the activity of GraS.^213^ Deletion of the guard loop of VraG also leads to knock-on effects such as increased transcription of the MprF gene, which lysinylates phosphatidylglycerol and helps repel CAMPs from the cell membrane.^213^ The GraXRS system also affects regulation of the *dlt* operon, which is responsible for D-alanylation of wall teichoic acids, increasing the positive surface charge of the cell envelop and repelling CAMPs.^213^ CAMPs are currently under investigation as an alternative treatment strategy to traditional antibiotics.^214^ *S. epidermidis*, like other species, has intrinsic resistance to some CAMPs, in this case through the activity of GraXSR, and so understanding the mechanisms behind this recognition and repulsion of CAMPs may help develop synthetic CAMPs to overcome resistance.^215^

## Other regulators

### ClpP protease and ATPase chaperones (ClpX, ClpC)

ClpP and ClpX constitute an ATP-dependent protease-chaperone system in *S. epidermidis*^216^ that orchestrates controlled degradation of specific protein substrates, contributing to cellular homoeostasis.^217^ Under cellular stress (e.g. antibiotics, temperature, oxidative stress), ClpP expression is increased to manage the accumulation of misfolded proteins, contributing to cellular fitness and survival.^217^ ClpXP-mediated proteolytic regulation of stress response regulators enhances cellular resilience.^218^ Paradoxically, deletion of ClpX or ClpP alone improves stationary-phase fitness because the survival of individual cells in stationary phase is enhanced in the absence of ClpXP-dependent degradation of misfolded proteins.^219^ Deletion of ClpX in *S. aureus* also enhances survival at high temperatures due to the compensatory role of the heat shock-induced chaperone ClpC,^220^ whose role in *S. epidermidis* remains largely unexplored, although it is present in the genomes of many clinical and animal isolates.^196, 221^

ClpP positively regulates biofilm formation, especially primary attachment and PIA production (Figure 3), impacting virulence in a rat IV-catheter infection model.^216^ Mutations in *spx*, encoding an RNA polymerase binding protein conserved in the Bacillota, reversed growth and competence defects caused by ClpX or ClpP mutations in *Bacillus.*^222^ Deletion of ClpP appears to have no direct effect on *ica* operon transcription. However, because ClpP controls the activity of Spx by proteolytic degradation (Figure 3),^223^ mutation of *clpP* is accompanied by downregulation of the *ica* operon, implicating an unidentified intermediate factor, possibly another ClpP protein target, that neutralizes the negative regulatory effect of Spx on *ica* transcription.^216,224^ Downregulation of ClpP by a functional *agr* system is also consistent with *agr-*mediated negative regulation of biofilm formation, favoring release of planktonic cell for dissemination.^216, 224^ This flexible regulation of a key virulence factor highlights the capacity of *S. epidermidis* to respond to environmental and population density cues to ensure persistence within the host.

## Phenotypic variation and IS256

IS*256* belongs to the IS6 family of insertion sequences and is characterized by its relatively small size (1.2 kb) and the presence of terminal inverted repeats (IRs). These IRs facilitate its transposition within the bacterial genome, allowing IS*256* to introduce genetic variability within *S. epidermidis* populations, including gene inactivation, up- or down-regulation of gene expression and chromosomal rearrangements.^225^ This genetic diversity can lead to variations in virulence factor expression and pathogenicity amongst isolates.

Compared to *icaADBC*^*+*^ commensal strains, clinical isolates with *icaADBC* are considerably more likely to carry IS*256* (85% vs. 15%).^226^ IS*256* is known to modulate the biofilm phenotype and can cause a switch from PIA-dependent to PIA-independent biofilm when inserted into the *icaC* locus,^227^ as well as decreasing *ica* operon expression and PIA production when inserted into the *rsbU* and *sarA* loci.^180^ An important aspect of IS*256* phenotypic switching is that it is reversible, as the insertion sequence can be subsequently excised from the gene, leaving an intact wild-type locus.^228^ This clean excision is a rare event, however, and appears to be independent of IS*256* transposition, which normally leaves 8-bp target site duplications (TSDs).^228^

The presence of IS*256* is considered a virulence marker,^229, 230^ as IS*256* is found more frequently in clinical isolates (up to 80%) of *S. epidermidis* compared to community isolates (13%) from healthy volunteers.^231^ Isolates from sequence type 2 (ST2) have a high rate of IS*256* carriage, up to 100%.^232^ ST2 is a widely distributed sequence type that is commonly found in clinical infections, especially catheter infections^231^ and is prevalent in hospital environments.^233^ In prosthetic joint infections, up to 83% of isolates show the presence of IS*256*, compared to just 4% of commensal isolates.^234, 235^

IS*256* is frequently associated with the acquisition and dissemination of antibiotic resistance genes within *S. epidermidis* populations. Isolates containing IS*256* are more likely to exhibit resistance to multiple antibiotics,^236^ including β-lactams and aminoglycosides.^226^ IS*256* presence is also correlated with carriage of *mecA.*^237^ Whilst multiple copies of IS*256* are typically distributed throughout the *S. epidermidis* genome, it is also found as a component of transposon Tn*4001*, which confers aminoglycoside resistance.^238^

A striking example of the clinical significance of genomic rearrangements induced by IS*256* was reported in a case of recurrent meningitis, in which a switch from a biofilm-positive to biofilm-negative phenotype was associated with IS*256* translocation and large chromosomal rearrangements.^225^ The ability of a single strain to rapidly adapt within the host gives it a clear fitness advantage, particularly when host immune factors increase selective pressure. Furthermore, several studies have suggested that IS*256* carriage is a more predictive indicator of *S. epidermidis* virulence than the presence of *icaADBC*, supporting the idea that *S. epidermidis* virulence is also linked to its capacity for adaptation through establishment of genetically diverse subpopulations.^226, 229, 234, 239^

## Host-*S.* *epidermidis* interactions

*S. epidermidis* must modulate the innate immune response to facilitate its persistence. Moreover, the host response to *S. epidermidis* as a commensal organism and as a pathogen can vary significantly in response to the location and strain, as outlined in several recent reviews.^3, 8, 16, 240^

### Recognition and response

Identifying pathogens involves initial recognition of bacterial components by pattern recognition receptors (PRRs) such as Toll-like receptors (TLRs) and nucleotide-binding oligomerization domain (NOD)-like receptors on epithelial cells, macrophages, dendritic cells, and neutrophils^241, 242^. Following recognition, signalling pathways are triggered, leading to production of pro-inflammatory cytokines, chemokines, and AMPs that induce chemotaxis of immune cells and result in phagocytosis and bacterial killing.^243^ *S. epidermidis* activates different PRRs depending on its cell wall components and secreted toxins, such as lipoteichoic acid (TLR2), peptidoglycan (TLR2 and NOD2), lipoproteins (TLR1/TLR2 and TLR2/TLR6), and PSMs (TLR2 and TLR6).^244–250^

Notably, *S. epidermidis* can inhibit TLR signalling, as is seen on the skin surface where it dampens TLR2 response in keratinocytes and reduces TLR3-mediated inflammation following skin injury.^245^ Along with the inhibition of TLR2, *S. epidermidis* can also induce the expression of anti-inflammatory cytokines, such as IL-10 and TGF-β, and regulatory T cells that suppress the inflammatory response and promote tolerance to the commensal bacteria, discussed in further detail below.^251–253^ Strains isolated from atopic dermatitis are more likely to cause a pro-inflammatory cytokine response in an *in vitro* model than strains from the skin of healthy patients, highlighting the complex role of *S. epidermidis* in epithelial immunity.^254^

### Immune evasion

Neutrophils/polymorphonuclear leukocytes (PMNs) form an important part of host defence against *S. epidermidis*. Biofilm is particularly associated with downregulation of pro-inflammatory cytokines, allowing infection to persist undetected.^255^ Production of PIA shields *S. epidermidis* from PMN phagocytosis, although PIA-independent biofilm can also impede effective opsonization by antibodies and complement components.^74, 256, 257^ Evasion of PMNs by *S. epidermidis* is also associated with repulsion and proteolysis of AMPs by GraRS and SepA respectively, as well as biofilm-mediated shielding of cells from IgG deposition.^137, 258^ Broader evasion of the complement system also contributes to *S. epidermidis* survival and persistence in the host^259^. Phagocytic killing of staphylococci is mediated primarily by the C3b and C5a opsonins,^259^ and whilst *S. epidermidis* can activate the complement system, effective opsonization by C3b is impeded in a biofilm.^258^

## *S.* *epidermidis* as a beneficial member of the skin microflora

### Competitive exclusion

*S. epidermidis agr* signals can interfere with the *S. aureus agr* system, resulting in downregulation of virulence factors required for *S. aureus* to establish infection.^165, 260^ Agr type I and IV *S. epidermidis* strains from patients with atopic dermatitis exhibited inhibitory effects on the expression of *S. aureus* virulence factors *in* *vitro*, and reduced *S. aureus* colonization of the skin in a mouse model.^261^

*S. epidermidis* produces several antimicrobial peptides that inhibit the growth of *S. aureus*, including bacteriocins. Bacteriocins are antimicrobial peptides synthesized by the ribosome and typically target species that are closely related to the producer,^262^ in order to stave off competition for a particular niche. For example, 96% of nasal *S. epidermidis* isolates produce bacteriocins.^263^ As a result, many *S. epidermidis* bacteriocins are active against *S. aureus* as well as other *S. epidermidis* and CoNS strains. *S. epidermidis* is known to produce the bacteriocins pep5,^264^ epidermin,^265^ epilancin K7^264^ epilancin 15×,^266^ nukacin IVK45^263^ and epicidin 280.^267^ A skin isolate of *S. epidermidis* has been described that produces epidermicin NI01, a plasmid-encoded bacteriocin.^268^ Plasmid-encoded nukacin IVK45 shows sequence similarity to genes in other staphylococcal species, indicating horizontal gene transfer of bacteriocins between species.^263^

On healthy skin, *S. epidermidis* is found to inhibit colonization with harmful *Cutibacterium acnes*, including selective activity against *C. acnes* strains associated with acne.^269^ These strains of *S. epidermidis* are typically not from the sequence types associated with infection^269^. Co-culture experiments with *C. acnes* and a representative strain of *S. epidermidis* showed that EpiA (precursor peptide for epidermin, which is known to inhibit survival of *C. acnes*) and PSMβ expression increased upon co-culture with *C. acnes.*^269^

*S. epidermidis* can also interfere with the virulence of other staphylococcal species. Co-culture of *S. epidermidis* with *S. aureus* results in SaeRS-dependent downregulation of the *S. aureus hla-*encoded haemolysin,^270^ via a mechanism involving an unidentified non-proteinaceous factor present in culture supernatants.^270^ The secreted serine protease Esp, which as noted above processes Aap and degrades complement protein C5, also impairs *S. aureus* biofilm production^271^ and can decolonise *S. aureus* from the nasal passages.^131^ Furthermore, Esp is also involved in nutrient acquisition via the breakdown of proteins into glutamate, which can be used by *S. epidermidis* as an energy source.^132^ Esp highlights the overlapping functions of *S. epidermidis* persistence/colonisation and virulence factors.^80^

### Immunomodulation

Gamma delta T-cells (GD T-cells) found in the epithelium are activated by the presence of *S. epidermidis*, leading to increased expression of *p*-2, an antimicrobial effector protein that kills *S. aureus* by forming pores in the bacterial membrane of internalised cells, as well as the TNF family transmembrane protein Fas Ligand and the pore-forming toxin granulysin.^272^ *S. epidermidis* LTA can activate Toll-like receptor 2 (TLR2) on keratinocytes that have been triggered to induce inflammation via Toll-like receptor 3 (TLR3) in response to skin injury.^272^ This interaction triggers an anti-inflammatory response through the NF-κB signalling pathway,^273^ reducing proinflammatory cytokines like TNF and IL-6 production, as well as inducing the production of AMPs.^245, 274^ Similarly, LTA can induce production of IL-6, IL-1β and TNF and elevate nitric oxide levels in macrophage cells lines while also stimulating an IgG response in patients with *S. epidermidis* device-associated infections.^275^ Commensal colonisation with *S. epidermidis* has been shown to induce a specific and co-ordinated dendritic cell response that enhances IL-17A^+^ CD8^+^ T-cell migration to the skin, which is associated with improved barrier function.^247^ These findings indicate that *S. epidermidis-*mediated modulation of the immune system is associated with both benefit and harm to patients, in keeping with its ability to switch from a protective member of the skin microflora to a pathogen. Indeed, the beneficial effects of *S. epidermidis* on keratinocyte and GD T-cell mediated-responses in the outermost epithelial cells of the skin contrasts with the deleterious impact on macrophage-mediated responses, which are more commonly detected in the underlying dermis following skin breach.^276^

### Maintenance of skin barrier function and homeostasis

*S. epidermidis* plays a role in maintaining the skin’s hydration and preventing skin disorders like atopic dermatitis by regulating skin barrier function.^277^ Production of the sphingomyelinase Sph, a hydrolase that breaks down sphingolipids onto ceramides and phosphocholine, benefiting the organism, protecting the skin from dehydration and promoting healthy turnover of skin cells.^3, 158^ Using a mouse model, butyrate, which is a fatty acid metabolite produced by *S. epidermidis*, was shown to protect the epidermis from IL-6 production induced by damaging UVB light.^278^ *S. epidermidis* also produces trace amines from aromatic amino acids (AAAs) found in high concentrations in skin.^279^ Whilst *S. epidermidis* does not appear to utilise AAAs in its own metabolism, excretion of trace amines by SadA-mediated decarboxylation of AAAs accelerates wound healing in a mouse model, via a mechanism associated with internalisation of the bacteria into host cells (offering protection from the host immune system) and inhibition of epinephrine production by keratinocytes (which negatively impacts wound healing by inhibiting cell migration).^279^ However, it is important to note that whilst *S. epidermidis* may provide benefits for the host, the function of these interactions is clearly to ensure the survival of the bacteria. Persistence in the host may be benign until circumstances allow for the establishment of infection.

## Diagnostics

Diagnostics for invasive *S. epidermidis* isolates responsible for infection is complicated by the ubiquitous presence of this organism on skin and mucous membranes. Efforts to evaluate the significance of a single positive blood culture of *S. epidermidis* as indicative of bacteraemia when concurrent with clinical symptoms have shown that whilst consecutive positive cultures is less prone to false positives, the incubation time between from culture initiation to culture positivity also predictive of bacteraemia and requires a shorter delay before diagnosis and treatment.^280–282^ For patients with symptoms of catheter-related sepsis, the gold standard is isolation of the same strain from catheter tips and blood cultures.^283^ In the case of PJIs, such as elbow arthroplasty, up to 78% of joint aspirations fail to yield a positive culture, which may be due to difficulties in culturing bacteria from a biofilm, whilst the current gold standard of biopsy and culture is prone to false positives (7.5%) and may result in overtreatment.^284–286^ Non-culture-based methods such as Raman spectroscopy are faster than traditional culture-based methods and show promising results for diagnosis of bone graft infections.^287^ Quantitative PCR methods have thus far been hindered by difficulty in finding suitable markers which are both actively transcribed during infection and conserved amongst isolates, whilst minimising the risks of false positives from non-invasive commensal strains.^288^ Multiplex real time PCR is capable of distinguishing *S. epidermidis*, MRSE, *S. aureus* and MRSA.^289^

Automated identification systems such as the bioMérieux Vitek 2, BectonDickinson Phoenix and Siemens Microscan assign a species level identification based on the results of biochemical tests.^290^ These automated systems reduce the time and workload required for bacterial identification, and can show high accuracy for *S. epidermidis*, though other CoNS species are not so easily identified.^291^ However, unlike real time PCR methods, these systems still require preculture of the isolates.^290^ Matrix-assisted laser desorption/ionisation time-of-flight mass spectrometry (MALDI-TOF MS) systems facilitate rapid species level identification of CoNS.^292–295^ Whilst most typically require growth of the isolate on agar plates prior to analysis, MALDI-TOF MS can be carried out directly on the clinical specimen, for example synovial fluid directly from PJIs, with better sensitivity than culture-based methods.^296, 297^ Attempts to use MALDI-TOF MS to differentiate MRSE from MSSE have been only partially successful, as the results are prone to false negatives.^298, 299^ A small study also showed that MALDI-TOF MS could potentially differentiate between biofilm-producing and non-biofilm producing strains.^300^

### Current antimicrobial treatment strategies

In the treatment of *S. epidermidis* infections, antibiotic selection is predicated upon the location of infection, antibiotic susceptibility profile, and the presence of prosthetic devices.

For bloodstream infections and catheter-related infections, vancomycin remains the gold standard due to its efficacy against MRSE, with β-lactams such as nafcillin and oxacillin also considered based on the resistance profile of the pathogen.^17, 301^ For ocular infections, fluoroquinolones, such as moxifloxacin or levofloxacin, are frequently utilized owing to their good penetration into ocular tissues and broad-spectrum activity.^302^ In the case of endophthalmitis, which can cause severe visual loss if time to treatment is delayed and of which *S. epidermidis* is a leading cause of infection, intravitreal injection of vancomycin can be used.^303^ Device-related infections, particularly those involving biofilm formation, often require a combination of surgical intervention and antibiotic therapy, with rifampicin combined with a glycopeptide (e.g. vancomycin) or a lipopeptide (e.g. daptomycin) recommended to eradicate biofilm-associated bacteria without promoting the emergence of rifampicin-resistant staphylococci.^301, 304, 305^

## Antimicrobial resistance

### Emergence of methicillin-resistant *S.* *epidermidis* (MRSE)

The emergence of MRSE mirrors the emergence and spread of MRSA. Acquisition of *mecA*, reduces susceptibility to methicillin and a broad spectrum of β-lactam antibiotics. The *mecA*-encoded penicillin-binding protein 2a (PBP2a) is less susceptible to binding by all β-lactams, including β-lactamase-resistant derivatives such as methicillin, cloxacillin, flucloxacillin, and oxacillin.^196, 306, 307^ The 13 known variants of SCC*mec*, which carries the *mecA* gene, are differentiated based on the composition of their cassette chromosome recombinase (*ccr*) and *mec* gene complexes.^308–311^ Types I-VI, including various subtypes have been described in *S. epidermidis*, with type IV generally being most frequent.^312–319^ Through the action of the recombinase genes in SCC*mec*, the cassette can be excised from the genome and transferred between isolates and between species, and there is evidence that *S. epidermidis* can function as a reservoir for methicillin resistance that can be passed on to other species, including *S. aureus.*^320–322^ The prevalence of MRSE has increased drastically in frequency over the past two decades and is more common in nosocomial isolates.^323^ In certain populations, such as neonates with bloodstream infections, methicillin resistance rates can reach 100%.^324, 325^

### Fluoroquinolone resistance

Infections caused by fluoroquinolone-resistant *S. epidermidis*, typically associated with point mutations in DNA gyrase (*gyrA*, *gyrB*) and topoisomerase IV (*parC*, *parE*) genes,^326^ narrow the range of therapeutic options. Moreover, fluoroquinolone resistance is increasing in prevalence, with 23% to 67% of *S. epidermidis* isolates being resistant to ciprofloxacin,^327–329^ and up to 60%, 55%, and 27% of MRSE and MSSE intravitreal isolates exhibiting resistance to levofloxacin, moxifloxacin, and delafloxacin, respectively.^330^ Topical fluoroquinolone solutions used prophylactically for cataract surgery have been correlated with an increased prevalence of fluoroquinolone-resistant *S. epidermidis* strains, including potential within-host adaptation during a 14-day treatment timeframe,^331^ although transmission of resistant strains from outside the host remains more common.^332^

### Glycopeptide resistance

Whilst planktonically-growing strains of *S. epidermidis* are susceptible to glycopeptides like vancomycin and teicoplanin, biofilms exhibit high tolerance to these antibiotics.^333–335^ Heteroresistance in subpopulations of laboratory grown strains to vancomycin was first recorded in a *S. epidermidis* human isolate in 1996.^336, 337^ Distinguishing homogeneous resistance from heteroresistance is challenging, but even heteroresistant strains can cause recurrent infection following treatment with vancomycin.^338–341^ Heteroresistance in catheter-associated bloodstream infections is associated with poor response to glycopeptide treatment, resulting in persistent infection, prolonged hospitalization and death.^342^ *S. epidermidis* is more commonly resistant to teicoplanin than vancomycin^323, 343, 344^ and newer generation lipoglycopeptides, including dalbavancin, are currently very effective against *S. epidermidis in vivo* and against planktonic and biofilm *S. epidermidis* cells *in vitro*, extending therapeutic options even after vancomycin treatment failure.^345–351^ However, mutations in *walK*, the histidine kinase of the essential two-component signalling system WalKR involved in regulating cell-wall biosynthesis and turnover,^352^ can lead to dalbavancin heteroresistance in patient isolates during treatment.^353^

### Fusidic acid resistance

Resistance to fusidic acid is mediated by Fus family proteins (FusA, FusB, FusC, FusD, FusF) that interfere with antibiotic binding to the ribosome.^354, 355^ FusA, FusD, and FusF are responsible for intrinsic resistance in species including *S. saprophyticus* and *S. cohnii*. In *S. epidermidis*, the most reported Fus proteins are FusB and FusC.^356, 357^ FusB is often carried on mobile phage-related resistance islands that are inserted at *att* integrase sites in heterologous strains.^358, 359^ The capacity for inter-strain or inter-species transfer of *fus* genes raises concern, particularly as their carriage has a low fitness cost for staphylococci.^355, 360^

### Linezolid resistance

Resistance to the oxazolidinone drug linezolid can arise due to mutations in the 23S rRNA gene.^361^ Furthermore, methylation of the 23s rRNA A_2503_ residue by the *cfr-*encoded methyltransferase leads to resistance to oxazolidinones, phenicols, lincosamides, pleuromutilins, and streptogramin A via decreased antibiotic binding.^361^ Linezolid-resistant *S. epidermidis* has been implicated in several endemic outbreaks in clinical settings.^362–365^ Unlike 23S rRNA mutations, *cfr*-mediated resistance is carried on a plasmid with a low fitness cost to the host cell,^366–370^ although linezolid resistant isolates can possess both *cfr* plasmids and 23S rRNA mutations.^371^ First reported in a *S. sciuri* bovine isolate in 2000 and human staphylococcal strains in 2008, *cfr*-bearing plasmids may also carry resistance genes for macrolide-lincosamide-streptogramin B antibiotics (MLS_B_) and spectinomycin.^366, 372, 373^ Recent reports of MRSE carrying *cfr*-containing plasmids are particularly concerning, as all strains investigated belonged to ST2, a clonal lineage that is a frequent cause of nosocomial infections and is notable for its worldwide dissemination and multi-drug resistance characteristics.^374^ Furthermore, several *S. aureus* isolates from the same hospital contained highly similar plasmids, potentially because of transfer from *S. epidermidis.*^374^

### Macrolide resistance

Resistance to macrolides associated with cross-resistance to lincosamides and streptogramin B is primarily associated with *ermC.*^375^ Erythromycin resistance can be found in approximately 60–65% of *S. epidermidis* isolates.^376, 377^ *ermC* is found in up to 85% of constitutively-MLS_B_ resistant and 100% of inducible – MLS_B_ resistant *S. epidermidis* strains.^378^ Whilst ErmA, ErmB, and ErmC mediate resistance by methylation of 23S rRNA, other mechanisms of resistance to macrolides include drug efflux (associated with the *msr* genes) and phosphorylation of the antibiotics (associated with the *mph* genes).^378^

### Multi-drug resistance (MDR)

The prevalence of multi-resistant strains is increasing in clinical settings.^379–381^ MRSE already precludes the use of one of the best-tolerated classes of antibiotics, but highly-methicillin resistant strains can also exhibit high rates of resistance to erythromycin (up to 80%), tetracycline (33%), clindamycin (33%), and gentamicin (27%).^380^ Up to 90% of MRSE isolates can be multi-drug resistant (MDR), posing a significant challenge to treatment.^55, 382^ Amongst ocular isolates, MDR was detected more frequently in infection-related isolates than amongst non-infection-causing isolates, and MDR isolates were significantly more likely to be biofilm producers.^383^

#### S. epidermidis as a reservoir of resistance genes

Horizontal gene transfer of resistance cassettes such as SCC*mec* highlights the adaptability of *S. epidermidis* in both acquiring and disseminating resistance determinants.^360, 384–386^ Whilst the precise mechanism of SCC*mec* transfer *in vivo* remains unclear, *in vitro* experiments indicate that of *S. aureus* can be induced to take up SCC*mec* by transformation via upregulation of competence genes by two-component systems such as SrrBA.^387^ In a particularly striking illustration of inter-species transfer of resistance, sequence analysis of three staphylococcal strains (MRSA, MSSA, and MRSE) isolated from the same patient indicated that in-patient transfer of SCC*mec* from the MRSE strain to MSSA had occurred.^321^ Plasmid-mediated resistance to tetracycline, streptomycin, and erythromycin can also drive dissemination from *S. epidermidis* to other species.^360, 386^

## Prevention and treatment strategies

### Prevention

Prophylactic measures used as part of medical procedures include fluoroquinolones, known for their efficacy against MRSE, during ocular surgery,^388^ vancomycin in prosthetic joint revision surgery,^389^ and clindamycin, vancomycin (teicoplanin in Europe), cephalosporins or vancomycin in combination with rifampicin to prevent PJIs.^47, 390^ Cephalosporins and vancomycin are routinely used for cardiac device implantation, with *in vitro* data supporting their use in combination with rifampicin.^391^ The FDA has also approved the use of rifampicin/minocycline-impregnated mesh on cardiac implants as an additional preventive measure.^392^ Notably, antimicrobial catheter lock solutions (CLSs), which are traditionally used post-infection, also have prophylactic potential.^393^ Anticoagulants such as sodium citrate used in CLSs can be antimicrobial, although their use as prophylactics to prevent infection have not been standardized.^393^ Nevertheless, sodium citrate was shown to be the most effective CLS against *S. epidermidis*, while taurolidine demonstrated efficacy against *E. coli.*^392, 393^ However, the potential benefit of using antibiotics in CLSs must be weighed against the risk of promoting resistance, which has been observed with fluoroquinolones in ocular procedures.^394^ Furthermore, the choice of antibiotic may change over time, as is seen with low susceptibility to clindamycin among *S. epidermidis* and MSSA PJI isolates when this antibiotic is used prophylactically.^390^ In this regard, antimicrobial stewardship and susceptibility testing of the frequently isolated strains that cause infection are imperative for responsible use of prophylactic antibiotics.

### Antimicrobial coating of medical devices

An attractive approach to combatting infection of biomaterials exploits novel antimicrobial coatings to limit *S. epidermidis* colonization. Osteopontin, a bovine milk protein, dose-dependently blocks bacterial adhesion under shear flow, potentially targeting early biofilm formation adhesins.^395^ Polymer blends of polyvinyl chloride (PVC) and polystyrene-ethylene-butylene-styrene (SEBS), infused with the ionic liquid HdmimDMSIP, have activity against *S. epidermidis* and can be incorporated into devices such as catheter tips.^396^ Micro-fibrillated cellulose-based antimicrobial films applied to biomaterials are also efficacious against *S. epidermidis.*^397^ Cellulose-based hydrogels, incorporating crosslinking agents or ε-poly-L-lysine, also have potential as part of antimicrobial wound dressings.^398, 399^

Colloid materials, characterized by their small particle size and large surface area, offer promising avenues for designing surfaces that incorporate antimicrobial agents. Silver nanoparticles in chitosan nanogel, combined with ampicillin, were effective against oxacillin-resistant *S. epidermidis.*^400^ Colloidal suspensions with titanium-peroxy gel layers provide non-antibiotic solutions for combating implant infections.^401^

In dual-purpose biomaterial modification, coupling titanium with a cationic antimicrobial peptide exhibited significant bactericidal activity against *S. epidermidis* and improved osteoblast adherence.^402^ Coating titanium with carboxymethyl chitosan and bone morphogenic protein 2 also inhibited *S. epidermidis* colonization.^403^ Surface treatments must be carefully chosen due to varying efficacy against bacterial species and potential cytotoxicity to host cells.^400, 404–408^ For titanium implants, hydrophilicity plays a crucial role in minimizing *S. epidermidis* attachment, challenging the assumption that hydrophilicity alone predicts implant material efficacy.^409^ In vivo studies showed that hydrothermally and electrophoretically modified titanium surfaces had superior bacterial repulsion properties.^410^

### Quorum sensing inhibition

The therapeutic potential of quorum sensing inhibition, or “quorum quenching,” which generally centers around competitive inhibition using biosimilar molecules that interfere with autoinduction, the use of enzymes that degrade autoinducing peptides (AIPs), or interference with AIP synthesis was the subject of a recent commentary.^163^

### Vaccines and antibody therapy

Vaccination against an important commensal like *S. epidermidis* presents significant challenges and even if achieved may be potentially detrimental. However, anti-biofilm vaccines are an interesting possibility. Here, the choice of biofilm antigen will be critical key given that staphylococci express different surface epitopes under varying environmental and physiological conditions.^411, 412^ The antigen will need to be highly conserved, if not amongst all strains, then at least amongst strains associated with the target infection type, and the antigenicity of the bacterial epitope also needs to be considered.^411^

Early work using whole chloroform-inactivated *S. epidermidis* cells and cell-free filtrate confirmed the immunogenicity of these vaccines in mice, where both vaccines provided protection from *S. epidermidis* intraperitoneal challenge but did not offer significant protection from *S. aureus* mortality.^413^ Targeting SdrG, a fibrinogen-binding protein, with antibodies impaired *S. epidermidis* attachment to Fn-coated catheters and increased phagocytosis by macrophages.^414, 415^ Immunization with a subdomain of SdrG was also shown to induce antibody production and impaired infection by *S. epidermidis* O-47 in a mouse model.^416^ Another cell wall protein, SesC, showed promise as a vaccine candidate in mouse and rat models of infection.^417^ Other Ses proteins, including SesB, were also found to exhibit vaccine potential^418^ and immunization with the B-domain repeat of Aap resulted in decreased inflammation, impaired bacterial colonization and a significant IgG antibody response.^419^ The iron transport protein SitC has also been investigated as a vaccine epitope and more recently its *S. aureus* ortholog MntC, a manganese transporter, was shown to provide protection from both *S. aureus* and *S. epidermidis* infection in a murine infection model.^420–422^

Pagibaximab, a monoclonal antibody therapy that targets LTA, showed promise in preventing neonatal sepsis in phase II clinical trials.^423^ However, phase III trials showed that whilst the treatment was safe and well-tolerated, prevention of sepsis was unsuccessful.^424^

Unfortunately to date vaccines and antibody therapies to combat *S. epidermidis* infection and biofilm formation have not progressed beyond animal trials. Vaccination against more serious *S. epidermidis* infections, such as neonatal sepsis, is complicated by the immature immune response of neonates and infants that can fail to provide long-lasting memory *T*- and B-cell responses, as well as poor activity of antigen-presenting cells and lower IgG response compared to adults.^425^ Nevertheless, vaccine research will continue to be an important part of efforts to find improved ways to control *S. epidermidis* and other infections^426–429^

## Concluding remarks

The pathogenic success of *S. epidermidis* is deeply rooted not only in its biofilm-forming capacity but also in its resilience and remarkable genomic plasticity, which facilitates the acquisition of virulence determinants and rapid adaption to shifting environmental conditions. Research into CoNS infections lags *S. aureus*, and whilst *S. epidermidis* is the most studied of the CoNS species, there are still substantial gaps in our understanding of its virulence determinants, resistance modalities, and host–pathogen interactions. Recognition of the importance of CoNS as pathogens is imperative to maintain and expand the global research base in this field. Furthermore, our understanding of CoNS virulence would be improved if *S. aureus* investigators extended their research, where appropriate, to include comparative studies with *S. epidermidis* or other CoNS.

The pathogenicity of *S. epidermidis* is unique in several ways: *i*. the persistence factors that make *S. epidermidis* such an effective colonizer of the harsh epithelial environment and how these are redeployed to cause disease, *ii*. the role of genomic flexibility in the success of *S. epidermidis* as a pathogen, and *iii*. the ever-increasing emergence of multidrug resistant strains. Regulation of the major virulence determinant in *S. epidermidis*, biofilm, is significantly different to *S. aureus*, and further research is needed to fully understand this in the context of the distinct lifestyle of this pathogen and its interactions with the host including opportunistic infections. To revisit the central question of this review: are we overlooking important disease determinants when *S. epidermidis* is overlooked as a “common commensal” and not a ubiquitous opportunistic pathogen? The research literature focused on *S. epidermidis* in recent decades reflects a gradual shift in how this organism is viewed, both as an important commensal for cutaneous health and a complex pathogen capable of causing significant harm to vulnerable individuals. Taking a holistic view continues to provide a more complete understanding of the lifestyle, host–microbe interactions and virulence of *S. epidermidis*.

## Data Availability

The figures in this review are openly available in Figshare (DOIs: 10.6084/m9.figshare.25663989 (Figure 1); 10.6084/m9.figshare.25092626 (Figure 2) and 10.6084/m9.figshare.25092623 (Figure 3).

## Notes

All authors agree to be accountable for all aspects of the work.
